# Interactions between staphylococcal enterotoxins A and D and superantigen-like proteins 1 and 5 for predicting methicillin and multidrug resistance profiles among *Staphylococcus aureus* ocular isolates

**DOI:** 10.1371/journal.pone.0254519

**Published:** 2021-07-28

**Authors:** Min Lu, Jean-Marie Parel, Darlene Miller

**Affiliations:** 1 Department of Public Health Sciences, Miller School of Medicine, University of Miami, Miami, FL, United States of America; 2 Department of Ophthalmology, Ophthalmic Biophysics Center, Bascom Palmer Eye Institute, University of Miami Miller School of Medicine, Miami, FL, United States of America; 3 Department of Ophthalmology, Ocular Microbiology Laboratory, Bascom Palmer Eye Institute, University of Miami Miller School of Medicine, Miami, FL, United States of America; Suez Canal University, EGYPT

## Abstract

**Background:**

Methicillin-resistant *Staphylococcus aureus* (MRSA) and multidrug-resistant (MDR) *S. aureus* strains are well recognized as posing substantial problems in treating ocular infections. *S. aureus* has a vast array of virulence factors, including superantigens and enterotoxins. Their interactions and ability to signal antibiotics resistance have not been explored.

**Objectives:**

To predict the relationship between superantigens and methicillin and multidrug resistance among *S. aureus* ocular isolates.

**Methods:**

We used a DNA microarray to characterize the enterotoxin and superantigen gene profiles of 98 *S. aureus* isolates collected from common ocular sources. The outcomes contained phenotypic and genotypic expressions of MRSA. We also included the MDR status as an outcome, categorized as resistance to three or more drugs, including oxacillin, penicillin, erythromycin, clindamycin, moxifloxacin, tetracycline, trimethoprim-sulfamethoxazole and gentamicin. We identified gene profiles that predicted each outcome through a classification analysis utilizing Random Forest machine learning techniques.

**Findings:**

Our machine learning models predicted the outcomes accurately utilizing 67 enterotoxin and superantigen genes. Strong correlates predicting the genotypic expression of MRSA were enterotoxins A, D, J and R and superantigen-like proteins 1, 3, 7 and 10. Among these virulence factors, enterotoxin D and superantigen-like proteins 1, 5 and 10 were also significantly informative for predicting both MDR and MRSA in terms of phenotypic expression. Strong interactions were identified including enterotoxins A (*entA*) interacting with superantigen-like protein 1 (*set6-var1_11*), and enterotoxin D (*entD*) interacting with superantigen-like protein 5 (*ssl05/set3_probe 1*): MRSA and MDR *S. aureus* are associated with the presence of both *entA* and *set6-var1_11*, or both *entD* and *ssl05/set3_probe 1*, while the absence of these genes in pairs indicates non-multidrug-resistant and methicillin-susceptible *S. aureus*.

**Conclusions:**

MRSA and MDR *S. aureus* show a different spectrum of ocular pathology than their non-resistant counterparts. When assessing the role of enterotoxins in predicting antibiotics resistance, it is critical to consider both main effects and interactions.

## Introduction

Multidrug resistance has been increased globally that is considered a public health threat. Several previous studies revealed the emergence of multidrug-resistant (MDR) bacterial pathogens from different origins, especially birds, animals, and food chains which may be transmitted to human consumers resulting in severe illness [1–8]. As a ubiquitous Gram-positive bacterium, *Staphylococcus aureus* is a leading cause of ocular morbidity and blindness worldwide [9]. Infections include blepharitis (lids), conjunctivitis (conjunctiva), keratitis (cornea), endophthalmitis (intraocular fluids), and dacryocystitis (lacrimal system). Methicillin-resistant *S. aureus* (MRSA) is well recognized as getting impassable toward numerous antibacterial specialists from the efficacy of infection treatments in the crisis center [10–13]. According to the infectious keratitis survey from the American Society of Cataract and Refractive Surgery in 2008, MRSA is the most common pathogen causing infections after laser-assisted in situ keratomileusis [14]. Hence, detecting strong correlates and interactions for predicting methicillin and multidrug resistant status could help us understand the prevalence of *S. aureus*, give essential insights into ocular pathology, and provide information for drug development against *S. aureus* ocular infection.

Recognizing the virulence factors of methicillin and multidrug resistant *S. aureus* are essential in developing preventive measures for ocular infectious diseases. The gene encoding the penicillin-binding protein 2a or 2′ (PBP2a or PBP2′) (*mecA*) was found to be integrated into the chromosomal element (SCC*mec*) of MRSA [15]. *S. aureus* isolates with the *mecA* gene are more likely to be MDR and difficult to treat. Researchers have studied the association between MRSA and cytolysins (*α*–toxin, *γ*–toxin, Panton-Valentine leukocidin (PVL) [16–19]. The associations between *mecA* and staphylococcal superantigens and enterotoxins are still ambiguous. MRSA could be associated with superantigens such as staphylococcal enterotoxin [SE] B or C [20], and staphylococcal enterotoxin-like [SE-l] Q [19]. Enterotoxin gene cluster types *egc1, egc2* and *egc3* could also play a role in this association since they are correlated with enterotoxin and enterotoxin-like gene profiles in human nasal carriage and animal isolates of *Staphylococcus aureus* [21]. However, few studies focused on these virulence factors for *Staphylococcus aureus* ocular infection. Moreover, statistically the role of each virulence factor was often assessed in a univariate fashion using separate Chi-square test or Fisher’s exact test for each factor [22–25], without adjustment of other factors or consideration of interactions between these virulence factors. Given the high correlations between these virulence factors, it is critical to include as many staphylococcal superantigens and enterotoxins as possible in a unified model to examine the most significant ones.

One statistical challenge for including all the virulence factors in a classification model is the curse of dimensionality. For example, staphylococcal enterotoxins are members of a protein family of more than 20 different staphylococcal exotoxins sharing several biological activities and structural features [9, 26, 27]. We want to examine both the main and the interaction effects for many virulence genes. Moreover, we wonder if we can predict MRSA accurately in a classification model after adding as many staphylococcal superantigen and enterotoxin genes as possible as predictors since high prediction accuracy indicates systematic differences in MRSA and methicillin-susceptible *S. aureus* (MSSA) ocular pathologies. We also wonder if the predictors are also informative for distinguishing MDR status. Clinically this is important because it will allow us to determine the superantigen profile of persistent strains. In addition, high accuracy of the prediction models would potentially enable the routine application of antimicrobial susceptibility testing to prevent the emergence of antibiotic-resistant strains of potential public health concern, and informative virulence genes might be chosen for tracing the sources of infection in ocular infection. For this purpose, we adopted an ensemble machine learning classifier, Random Forest [28], to predict MRSA and MDR *S. aureus*, which over the last twenty years has become widely used for predictions in cardiovascular disease, cancer, HIV, and other non-medical endeavors [29–33]. The objective of this study is to identify virulence factors of staphylococcal superantigens and enterotoxins that are most important for predicting the risk of methicillin and multidrug resistance, and to demonstrate how these informative virulence factors differentiate the predicted probability or risk score of MRSA and MDR *S. aureus* ocular infection.

## Methods

This was a retrospective study of the molecular characteristics and interactions of *S. aureus* isolates recovered from ocular samples collected between January 2014—December 2019. Institutional review board approval was obtained from the University of Miami Miller School of Medicine Sciences Subcommittee for the Protection of Human Subjects and the research followed the tenets of the Declaration of Helsinki (IRB Protocol Study ID #20070960). All patients were informed and consented in writing. The Ocular Microbiology Department’s Isolate database was searched to identify, non-consecutive, de-identified (no patient information) *S. aureus* isolates recovered from patients presenting with culture proven *S. aureus* ocular infections. No patient data was included or available for the study.

We used a DNA microarray to characterize the enterotoxin and superantigen gene profiles [34] of 98 *S. aureus* isolates collected from common ocular sources. Seventy-six of these were from the cornea. There is a combination of 64 MRSA and 34 MSSA, and 69 MDR and 29 non-multidrug resistant (NMDR) isolates in terms of phenotypic characterization. MRSA status was determined through the E tests, cefoxitin screen and automated system. MDR status was defined as resistance to three or more drugs, including oxacillin, penicillin, erythromycin, clindamycin, moxifloxacin, tetracycline, trimethoprim-sulfamethoxazole (SXT), and gentamicin. The genotypic characterization of MRSA produced 56 *mecA*-positive and 42 *mecA*-negative isolates. The *mecA* gene was not detected in eight isolates that were categorized as MRSA in phenotypic assays. The difference is due to varied test methods, and microarray was done after the phenotypic assays—hence freezing may impact the stability of *mecA*. We include 21 enterotoxin genes, 45 staphylococcal superantigens-like genes, and one variable indicating isolate sources to predict MRSA and MDR statuses. In total, we have 67 predictors.

### Statistical analysis

Random Forest [28] model was applied for the prediction of outcome, a modern machine learning technique that permits exploration of nonlinear, complex interrelationships. It has been utilized to explore a large number of predictors and identify replicable sets of risk factors [35–38]. The Random Forest technology is related to recursive partitioning and classification tree analyses, wherein the variables that are most related to an outcome of interest are first optionally split to improve prediction, followed by more and more splits to create a tree. A single tree is inherently unstable with large prediction variance. To overcome this instability, a forest of trees is “grown” from bootstrap samples of the original dataset (sampling with replacement until a dataset of equal size is generated; an average of 37% of the data will not be sampled, which is referred as out-of-bag data), permitting an ensemble average to be calculated across the individual trees [28]. Because this method is completely nonparametric without any restrictive underlying model assumptions, complex relationship and interactions among variables can be robustly accounted for.

#### Classification analyses

Classification analysis was applied to two statuses, MRSA versus MSSA, and NMDR versus MDR, using the Random Forest classification model. Estimated variable importance (VIMP) of each predictor [28, 39] is adopted, which utilizes a prediction-based approach by estimating prediction error attributable to the predictor, evaluated as the cross-validated misclassification error estimated via the out-of-bag data. The VIMP can be interpreted as the increase in the misclassification error when the corresponding predictor is randomly permutated into a noise variable. Positive VIMP values identify variables that are predictive after adjusting for all the other variables. For example, a VIMP of 4.29% indicates that a variable improves by 4.29% the ability of the model to classify the status of the outcome. Negative VIMP values indicate “noisy” variables that degrade model performance. Standard errors and *P* values are generated by a delete-*d*-jackknife procedure [39]. Strong correlates were based on an *α* = 0.05 level confidence for VIMP. Variables with positive VIMP estimates whose *P* values less than 0.05 are selected as informative ones to predict the outcome.

Odds ratio is used to inform the direction of the associations between the outcome and the selected informative predictors, calculated for the negative and positive categories. The ambiguous category is not considered. Odds ratio > 1 means that positive isolate for the corresponding virulence gene is more likely to be MRSA or MDR, while Odds ratio < 1 indicates that positive isolate for the corresponding virulence gene is associated with MSSA or NMDR status. *P* values are calculated using Fisher’s exact test.

Random Forest model and odds ratio calculation were implemented in the open-source R software using the randomForestSRC [40] and epitools [41] R packages correspondingly. From the randomForestSRC R package, the function rfsrc was used for building the prediction model under default setting with 1000 trees; then inferences of VIMP were estimated using the function subsample. The default settings for this function were adopted with 1000 subsamples, except the ratio for subsampling approach which was increased from 10% (the default setting is the square root of the sample size) to 90% because of small sample size. The function oddsratio.fisher from epitools package was used to calculate odds ratios, confidence intervals and *P* values.

#### Partial plot

For the Random Forest classification analysis, partial dependence plots were used to visualize the variables’ impact on the predicted probabilities of the statuses of the outcome through mapping the marginal effect of the selected variable to uncovers the relationship [42–44], where predicted probability is defined as out-of-bag predicted probability adjusted by integrating out all variables other than the targeted variable of interest. The integration is approximated utilizing the data by averaging over variables, implemented through setting the partial parameter in the plot.variable function from the randomForestSRC R package.

## Results

### Summary of methicillin and multidrug resistant isolates

We summarized all the predictors stratified by MRSA and MDR statuses in Table 1. Pearson’s Chi-squared test is applied and implemented in the open-source R software for testing for independence. The percentages of positive isolates are listed in parentheses in Table 1. Most virulence genes display different percentages of positives between different MRSA and MDR statuses. For MRSA, such a difference is more likely to be significant for staphylococcal superantigen/enterotoxin-like genes than enterotoxin genes. For MDR *S. aureus*, enterotoxins C, D, and L levels are significantly different between NMDR and MDR isolates. As to isolate sources, fewer MRSA isolates were collected from the cornea (66.07% as 37 out of 56) than MSSA (92.86% as 39 out of 42, *p* = 0.021) in the microarray test of *mecA*. Such a difference was not significant for the phenotypic expression of MDR or MRSA.

**Table 1:** Distribution of virulence factors among methicillin and multidrug resistance profiles.

Virulence factor	Virulence gene	Negatives/Positives (percentage of positives)						Sig†
		Genotype		Phenotype		Phenotype		
		MSSA (n = 42)	MRSA (n = 56)	MSSA (n = 34)	MRSA (n = 64)	NMDR (n = 29)	MDR (n = 69)	
Enterotoxin A	*entA*	40/2 (5)	48/8 (14)	32/2 (6)	56/8 (12)	28/1 (3)	60/9 (13)	
Enterotoxin A, 320E	*entA (320E)*	42 (0)	56 (0)	34 (0)	64 (0)	29 (0)	69 (0)	
Enterotoxin A, N315	*entA (N315) / entP*	38/4 (10)	52/4 (7)	30/4 (12)	60/4 (6)	26/3 (10)	64/5 (7)	
Enterotoxin B	*entB*	37/5 (12)	49/7 (12)	29/5 (15)	57/7 (11)	26/3 (10)	60/9 (13)	
Enterotoxin C	*entC*	39/3 (7)	55/1 (2)	31/3 (9)	63/1 (2)	25/4 (14)	69/0 (0)	‡‡
Enterotoxin D	*entD*	40/2 (5)	46/10 (18)	33/1 (3)	53/11 (17)	29/0 (0)	57/12 (17)	‡
Enterotoxin E	*entE*	42 (0)	56 (0)	34 (0)	64 (0)	29 (0)	69 (0)	
Enterotoxin G	*entG*	19/23 (55)	21/35 (62)	16/18 (53)	24/40 (62)	12/17 (59)	28/41 (59)	
Enterotoxin H	*entH*	40/2 (5)	56/0 (0)	32/2 (6)	64/0 (0)	28/1 (3)	68/1 (1)	
Enterotoxin I	*entI*	19/23 (55)	21/35 (62)	16/18 (53)	24/40 (62)	12/17 (59)	28/41 (59)	
Enterotoxin J	*entJ*	37/5 (12)	46/10 (18)	30/4 (12)	53/11 (17)	26/3 (10)	57/12 (17)	
Enterotoxin K	*entK*	36/6 (14)	40/16 (29)	28/6 (18)	48/16 (25)	26/3 (10)	50/19 (28)	
Enterotoxin L	*entL*	39/3 (7)	55/1 (2)	31/3 (9)	63/1 (2)	25/4 (14)	69/0 (0)	‡‡
Enterotoxin M	*entM*	19/23 (55)	21/35 (62)	16/18 (53)	24/40 (62)	12/17 (59)	28/41 (59)	
Enterotoxin N	*entN (cons)*	19/23 (55)	21/35 (62)	16/18 (53)	24/40 (62)	12/17 (59)	28/41 (59)	
	*entN (other than RF122)*	19/23 (55)	21/35 (62)	16/18 (53)	24/40 (62)	12/17 (59)	28/41 (59)	
Enterotoxin O	*entO*	19/23 (55)	21/35 (62)	16/18 (53)	24/40 (62)	12/17 (59)	28/41 (59)	
*egc* cluster	*egc (total)*	19/23 (55)	21/35 (62)	16/18 (53)	24/40 (62)	12/17 (59)	28/41 (59)	
Enterotoxin Q	*entQ*	36/6 (14)	40/16 (29)	28/6 (18)	48/16 (25)	26/3 (10)	50/19 (28)	
Enterotoxin R	*entR*	38/4 (10)	46/10 (18)	30/4 (12)	54/10 (16)	26/3 (10)	58/11 (16)	
Enterotoxin U and/or Y	*entU*	19/23 (55)	21/35 (62)	16/18 (53)	24/40 (62)	12/17 (59)	28/41 (59)	
SSL protein 1	*set6-var1_11*	16/26 (62)	0/56 (100)	16/18 (53)	0/64 (100)	15/14 (48)	1/68 (99)	***+++‡‡‡
	*set6-var2_11*	27/14 (33)	54/0 (0)	19/14 (41)	62/0 (0)	15/13 (45)	66/1 (1)	***+++‡‡‡
	*set6-var1_12*	12/24 (57)	3/48 (86)	9/19 (56)	6/53 (83)	8/14 (48)	7/58 (84)	**+‡‡
	*set6-var2_12*	37/4 (10)	55/0 (0)	29/4 (12)	63/0 (0)	25/3 (10)	67/1 (1)	+
	*set6-var4_11*	6/36 (86)	1/55 (98)	6/28 (82)	1/63 (98)	6/23 (79)	1/68 (99)	*+‡‡
	*ssl01-RF122*	39/3 (7)	56/0 (0)	31/3 (9)	64/0 (0)	26/3 (10)	69/0 (0)	‡
	*ssl01/set6 (COL)*	32/10 (24)	35/21 (38)	27/7 (21)	40/24 (38)	25/4 (14)	42/27 (39)	‡
	*ssl01/set6 (Mu50+N315)*	17/15 (36)	1/34 (61)	17/10 (29)	1/39 (61)	17/8 (28)	1/41 (59)	***+++‡‡‡
	*ssl01/set6 (MW2+MSSA476)*	38/4 (10)	56/0 (0)	30/4 (12)	64/0 (0)	26/3 (10)	68/1 (1)	+
	*ssl01/set6 (MRSA252)*	38/4 (10)	56/0 (0)	30/4 (12)	64/0 (0)	25/4 (14)	69/0 (0)	+‡‡
	*ssl01/set6 (RF122)*	41/1 (2)	56/0 (0)	33/1 (3)	64/0 (0)	28/1 (3)	69/0 (0)	
	*ssl01/set6 (other alleles)*	34/8 (19)	55/1 (2)	26/8 (24)	63/1 (2)	20/9 (31)	69/0 (0)	*++‡‡‡
SSL protein 2	*ssl02/set7*	5/34 (81)	0/56 (100)	5/26 (76)	0/64 (100)	5/21 (72)	0/69 (100)	**+++‡‡‡
	*ssl02/set7 (MRSA252)*	11/7 (17)	15/0 (0)	7/7 (21)	19/0 (0)	7/7 (24)	19/0 (0)	**+++‡‡‡
SSL protein 3	*ssl03/set8_probe 1*	7/35 (83)	0/56 (100)	7/27 (79)	0/64 (100)	7/22 (76)	0/69 (100)	**+++‡‡‡
	*ssl03/set8_probe 2*	7/35 (83)	0/56 (100)	7/27 (79)	0/64 (100)	7/22 (76)	0/69 (100)	**+++‡‡‡
	*ssl03/set8 (MRSA252, SAR0424)*	40/2 (5)	56/0 (0)	32/2 (6)	64/0 (0)	27/2 (7)	69/0 (0)	
SSL protein 4	*ssl04/set9*	8/34 (81)	0/56 (100)	8/26 (76)	0/64 (100)	8/21 (72)	0/69 (100)	**+++‡‡‡
	*ssl04/set9 (MRSA252, SAR0425)*	34/7 (17)	53/0 (0)	26/7 (21)	61/0 (0)	21/7 (24)	66/0 (0)	**+++‡‡‡
SSL protein 5	*ssl05/set3_probe 1*	9/33 (79)	0/56 (100)	9/25 (74)	0/64 (100)	9/20 (69)	0/69 (100)	**+++‡‡‡
	*ssl05/set3 (RF122, probe-611)*	9/8 (19)	3/0 (0)	7/8 (24)	5/0 (0)	7/8 (28)	5/0 (0)	***+++‡‡‡
	*ssl05/set3_probe 2 (612)*	7/27 (64)	0/56 (100)	7/19 (56)	0/64 (100)	7/14 (48)	0/69 (100)	***+++‡‡‡
	*ssl05/set3 (MRSA252)*	35/7 (17)	56/0 (0)	27/7 (21)	64/0 (0)	22/7 (24)	69/0 (0)	**+++‡‡‡
SSL protein 6	*ssl06/set21*	24/18 (43)	36/20 (36)	19/15 (44)	41/23 (36)	18/11 (38)	42/27 (39)	
	*ssl06 (NCTC8325+MW2)*	17/20 (48)	30/21 (38)	12/17 (50)	35/24 (38)	11/13 (45)	36/28 (41)	
SSL protein 7	*ssl07/set1*	6/35 (83)	0/56 (100)	6/27 (79)	0/64 (100)	6/22 (76)	0/69 (100)	**+++‡‡‡
	*ssl07/set1 (MRSA252)*	5/2 (5)	0/0 (0)	5/2 (6)	0/0 (0)	5/2 (7)	0/0 (0)	**+++‡‡‡
	*ssl07/set1 (AF188836)*	24/5 (12)	41/0 (0)	17/5 (15)	48/0 (0)	14/5 (17)	51/0 (0)	*++‡‡‡
SSL protein 8	*ssl08/set12_probe 1*	7/35 (83)	0/56 (100)	7/27 (79)	0/64 (100)	7/22 (76)	0/69 (100)	**+++‡‡‡
	*ssl08/set12_probe 2*	7/35 (83)	0/56 (100)	7/27 (79)	0/64 (100)	7/22 (76)	0/69 (100)	**+++‡‡‡
SSL protein 9	*ssl09/set5_probe 1*	7/35 (83)	3/53 (95)	7/27 (79)	3/61 (95)	7/22 (76)	3/66 (96)	+‡‡
	*ssl09/set5_probe 2*	7/35 (83)	3/53 (95)	7/27 (79)	3/61 (95)	7/22 (76)	3/66 (96)	+‡‡
	*ssl09/set5 (MRSA252)*	35/7 (17)	56/0 (0)	27/7 (21)	64/0 (0)	22/7 (24)	69/0 (0)	**+++‡‡‡
SSL protein 10	*ssl10/set4*	11/31 (74)	0/56 (100)	11/23 (68)	0/64 (100)	11/18 (62)	0/69 (100)	***+++‡‡‡
	*ssl10 (RF122)*	33/4 (10)	49/0 (0)	25/4 (12)	57/0 (0)	19/4 (14)	63/0 (0)	+‡‡
	*ssl10/set4 (MRSA252)*	3/6 (14)	1/0 (0)	1/6 (18)	3/0 (0)	1/6 (21)	3/0 (0)	**++‡‡‡
SSL protein 11	*ssl11/set2 (COL)*	32/10 (24)	35/21 (38)	27/7 (21)	40/24 (38)	25/4 (14)	42/27 (39)	‡
	*ssl11+set2(Mu50+N315)*	28/14 (33)	22/34 (61)	25/9 (26)	25/39 (61)	22/7 (24)	28/41 (59)	*++‡‡
	*ssl11+set2(MW2+MSSA476)*	39/3 (7)	56/0 (0)	31/3 (9)	64/0 (0)	27/2 (7)	68/1 (1)	
	*ssl11/set2 (MRSA252)*	36/6 (14)	56/0 (0)	28/6 (18)	64/0 (0)	23/6 (21)	69/0 (0)	*++‡‡‡
SEL protein, second locus	*setB3*	7/35 (83)	0/56 (100)	7/27 (79)	0/64 (100)	7/22 (76)	0/69 (100)	**+++‡‡‡
	*setB3 (MRSA252)*	35/7 (17)	56/0 (0)	27/7 (21)	64/0 (0)	22/7 (24)	69/0 (0)	**+++‡‡‡
	*setB2*	7/35 (83)	0/56 (100)	7/27 (79)	0/64 (100)	7/22 (76)	0/69 (100)	**+++‡‡‡
	*setB2 (MRSA252)*	35/7 (17)	56/0 (0)	27/7 (21)	64/0 (0)	22/7 (24)	69/0 (0)	**+++‡‡‡
	*setB1*	2/38 (90)	0/56 (100)	2/30 (88)	0/64 (100)	2/25 (86)	0/69 (100)	+‡‡
Sources	*sources*	2/1/39/	16/0/36/	2/1/31/	16/0/44/	2/1/26/	16/0/49/	*
		0/0/0 (93)	1/1/2 (64)	0/0/0 (91)	1/1/2 (69)	0/0/0 (90)	1/1/2 (71)	

SSL indicates staphylococcal superantigen-like; SEL indicates Staphylococcal exotoxin-like; MSSA is short for methicillin-susceptible *S. aureus*, MRSA for methicillin-resistant *S. aureus*, NMDR for non-multidrug-resistant *S. aureus*, MDR for multidrug-resistant *S. aureus*. For isolate sources listed in the bottom, numbers of isolates are listed in the order of conjunctiva, conjunctiva/lids, cornea (percentage is listed in the parenthesis), lids, socket, and suture.

^†^ The association between the outcome and the virulence gene in category negative, positive, and ambiguous is tested through Pearson’s Chi-squared test. Sig indicates significant level according to *P* values: when the outcome is genotypic expression of MRSA, * for *p* ≤ 0.05, ** for *p* ≤ 0.01, *** for *p* ≤ 0.001; when the outcome is phenotypic expression of MRSA, + for *p* ≤ 0.05, ++ for *p* ≤ 0.01, and +++ for *p* ≤ 0.001; when the outcome is MDR, ‡ for *p* ≤ 0.05, ‡‡ for *p* ≤ 0.01, and ‡‡‡ for *p* ≤ 0.001.

* indicates the genotypic expression between MSSA and MRSA; + for the phenotypic expression between MSSA and MRSA; ‡ for the phenotypic expression between NMDR and MDR.

Two enterotoxins, *entA (320E)* and *entE*, contain no positive isolates, and therefore they are unable to predict any outcome. There are virulence genes with identical detection results, for example, enterotoxins M, N, and O. In order to reduce the dimension and collinearity of the classification model, when the values of a group of variables are the same, we only add one of them in the classification model whose result will represent the entire group. Variables with duplicated results are listed in S2 Table. There are 46 variables in each prediction model.

The Random Forest classification model predicts the outcomes accurately: the out-of-bag misclassification error is 23.47% for predicting the genotypic expression of MRSA, 17.35% for the phenotypic expression of MRSA and 14.29% for MDR *S. aureus*. The model predicting MDR *S. aureus* has the lowest misclassification error, indicating that superantigens and enterotoxins are more associated with MDR *S. aureus* than MRSA. As to the phenotypic expressions, we found that MRSA isolates are highly likely to be MDR with an infinite odds ratio (see S3 Table for the contingency table of the three outcomes). The genotypic expression of MRSA is also significantly associated with MDR status (odds ratio = 18.4, *p* <.001).

### Relationship of outcomes to important variables

#### Genotypic expression of MRSA

Variables of importance for predicting each outcome were identified by machine learning using Random Forest classification model. For the genotypic expression of MRSA, 16 variables are significant from its classification analysis, shown in Table 2. Strong correlates predicting MRSA include enterotoxin A, P, D, J, and R and staphylococcal superantigen-like proteins 1, 3, 5, 7, and 10. The full list of VIMP and odds ratios for the 67 predictors can be found in S1 Table. Isolate sources are not informative (see the bottom of S1 Table), which means that after adjusting for differences in virulence genes, isolate sources are balanced between MSSA and MRSA isolates. Univariately, none of the informative enterotoxins are significantly associated with MRSA. However, after adjusting for other variables, enterotoxin P positive indicates a higher probability of MRSA, as shown in the partial plot of *entA (N315) / entP* in subfigure A of S1 Fig. All the selected staphylococcal superantigen-like proteins are significantly associated with MRSA from Fisher’s exact test (see Table 2), except *ssl05/set3 (RF122, probe-611)* and *ssl07/set1 (MRSA252)*. After adjusting for other variables, *ssl05/set3 (RF122, probe-611)*-positive is associated with MRSA (subfigure B of S1 Fig) and *ssl07/set1 (MRSA252)* negative is associated with MRSA (subfigure C of S1 Fig). As to enterotoxins, the most informative virulence gene is *entD*, contributing 3.1% prediction accuracy (standard error (SE) = 0.98, *p* <.001). In other words, without taking into consideration *entD*, the prediction error or misclassification rate of the Random Forest model will increase from 23.47% to 26.57%. As to staphylococcal superantigen-like proteins, *ssl05/set3_probe 2 (612)* is the most informative (VIMP = 2.64, SE = 0.55, *p* <.001).

**Table 2:** Informative virulence genes for predicting methicillin and multidrug resistance profiles.

Virulence factor	Virulence gene	Fisher’s exact test		Random Forest				
		Genotype		Genotype			Phenotype	
		MRSA		MRSA			MRSA†	MDR†
		OR	95% CI	Est	SE	Sig	Sig	Sig
Enterotoxin A	*entA*	3.3	0.61–33.61	0.89	0.49	*		
Enterotoxin A, allele from strain N315 = enterotoxin P	*entA (N315) / entP*	0.73	0.13–4.2	1.09	0.52	*	+	
Enterotoxin D	*entD*	4.29	0.84–42.53	3.1	0.98	***	+++	‡‡‡
Enterotoxin J	*entJ*	1.6	0.45–6.51	0.77	0.32	**		‡
Enterotoxin R	*entR*	2.05	0.54–9.68	1.78	0.63	**		‡
Staphylococcal superantigen-like protein 1	*set6-var1_11*	Inf	7.39-Inf	1.8	0.41	***	+++	‡‡
	*set6-var2_11*	0	0–0.17	1.07	0.32	***	+++	
	*ssl01/set6 (MW2+MSSA476)*	0	0–1.1	0.14	0.06	**	++	
Staphylococcal superantigen-like protein 3	*ssl03/set8_probe 1*	Inf	2.12-Inf	0.07	0.04	*	+	
Staphylococcal superantigen-like protein 5	*ssl05/set3_probe 1*	Inf	3.06-Inf	0.35	0.16	*	++	‡‡‡
	*ssl05/set3 (RF122, probe-611)*	0	0–3.54	1.71	0.46	***	++	‡‡
	*ssl05/set3_probe 2 (612)*	Inf	2.71-Inf	2.64	0.55	***	+++	‡‡‡
	*ssl05/set3 (MRSA252)*	0	0–0.47	0.08	0.05	*	+	
Staphylococcal superantigen-like protein 7	*ssl07/set1 (MRSA252)*	0	0-Inf	0.18	0.1	*		
	*ssl07/set1 (AF188836)*	0	0–0.71	2.13	0.84	**		
Staphylococcal superantigen-like protein 10	*ssl10/set4*	Inf	4.11-Inf	0.41	0.15	**	++	‡‡

OR indicates odds ratio, which is used to indicate the direction of the correlation: if the value is larger than one, the virulence gene is more likely to be detected (as more positive isolates) in MRSA isolates; if the value is smaller than one, the virulence gene is less likely to be detected (as more negative isolates) in MRSA isolates. Inf indicates infinity value since some entry of the contingency table is zero.

Est indicates estimated VIMP from Random Forest model in terms of contribution to classification accuracy in percentage; SE indicates standard error; Sig indicates significant level according to *P* values: * for *p* ≤ 0.05; ** for *p* ≤ 0.01; *** for *p* ≤ 0.001.

^†^ Two Random Forest models were fitted to classify phenotypic expressions of MRSA and MDR statuses, and the significant level of estimated VIMP was used to mark the informative virulence genes: when the outcome is MRSA, + for *p* ≤ 0.05, ++ for *p* ≤ 0.01, and +++ for *p* ≤ 0.001 (see S4 Table for full list of informative predictors); when the outcome is MDR *S. aureus*, ‡ for *p* ≤ 0.05, ‡‡ for *p* ≤ 0.01, and ‡‡‡ for *p* ≤ 0.001 (see S5 Table for full list of informative predictors).

#### Phenotypic expression of MRSA

As mentioned before, eight isolates were categorized as MRSA in phenotypic assays but classified as *mecA*-negative in the microarray test. These eight isolates cause differences in the classification results between the genotypic and phenotypic expressions of MRSA. However, the informative virulence genes with large estimates of VIMP are still very similar. For example, *entD* and *ssl05/set3_probe 2 (612)* are the most informative genes for the genotypic expression of MRSA. For the phenotypic expression of MRSA, *entD* (VIMP = 2.82, SE = 0.83, *p*<.001) and *ssl05/set3_probe 2 (612)* (VIMP = 2.33, SE = 0.56, *p* <.001) are also the most informative genes. As shown in S4 Table, strong correlates predicting the phenotypic expression of MRSA include enterotoxins P and D and staphylococcal superantigen-like proteins 1, 3, 4, 5, 7, and 10. For staphylococcal superantigen-like proteins 3, 5 and 10, the informative genes are identical for predicting both the genotypic and phenotypic expressions of MRSA. Three virulence genes, *set6-var4_11, ssl01/set6 (Mu50+N315)* and *ssl04/set9*, are significantly informative for predicting the phenotypic expression of MRSA, but their effects for predicting the genotypic expression of MRSA are not significant. Four virulence genes, *entA, entJ, entR* and *ssl07/set1 (AF188836)*, are significantly informative for predicting the genotypic expression of MRSA, but their effects for predicting the phenotypic expression of MRSA are not significant. The contribution of these seven virulence genes on the prediction accuracy is limited, since their VIMP estimates are small.

#### Phenotypic expression of MDR *S. aureus*

For MDR *S. aureus*, enterotoxin D (*entD*, VIMP = 3.63, SE = 1.01, *p* <.001) and staphylococcal superantigen-like protein 1 (*ssl01/set6 (Mu50+N315)*, VIMP = 2.27, SE = 0.48, *p* <.001) are the most informative factors (see S5 Table). Eight virulence genes, *entD, entJ, entR, set6-var1_11, ssl05/set3_probe 1, ssl05/set3 (RF122, probe-611), ssl05/set3_probe 2 (612)* and *ssl10/set4*, are significantly informative for predicting both MDR *S. aureus* and the genotypic expression of MRSA. Nine virulence genes, *entD, set6-var1_11, set6-var4_11, ssl01/set6 (Mu50+N315), ssl04/set9, ssl05/set3_probe 1, ssl05/set3 (RF122, probe-611), ssl05/set3_probe 2 (612)* and *ssl10/set4*, are significantly informative for predicting the phenotypic expressions of both MDR and MRSA. Unlike MRSA, MDR status is associate with enterotoxins in a directly proportional relationship (see Table 1 for *entC, entD*, and *entL*). In Table 2, predictors that are also significantly informative for the phenotypic expressions of MDR and MRSA are marked in the last two columns: *entD, set6-var1_11, ssl05/set3_probe 1, ssl05/set3 (RF122, probe-611), ssl05/set3_probe 2 (612)*, and *ssl10/set4* are significantly informative for predicting all the outcomes. The outcomes are highly correlated. Therefore, the classification results are similar in terms of the sign of the VIMP estimates. However, the inferences could vary since the predictors correlate with each other, especially among virulence genes under the same category of virulence factor. The model predicting MDR *S. aureus* has the highest prediction accuracy: utilizing the combination of these 46 genes encoding staphylococcal enterotoxins and superantigen-like proteins, we can predict 85.71% *S. aureus* isolates correctly in terms of NMDR versus MDR status.

### Interactions between virulence factors

**Fig 1:**
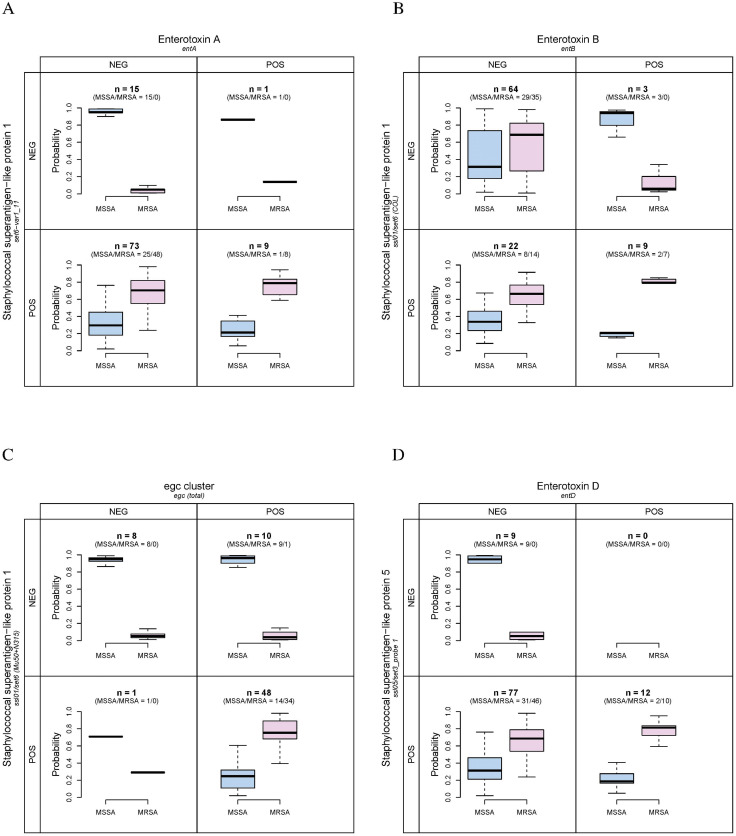
Random Forest estimated probabilities of MSSA and MRSA in terms of genotypic expression plotted against selected interactions between staphylococcal superantigens and enterotoxins. The numbers of detected MSSA and MRSA are listed in the parentheses. A: The interaction between *entA* and *set6-var1_11*. B: The interaction between *entB* and *ssl01/set6 (COL)*. C: The interaction between *egc (total)* and *ssl01/set6 (Mu50+N315)*. D: The interaction between *entD* and *ssl05/set3_probe 1*.

**Fig 2:**
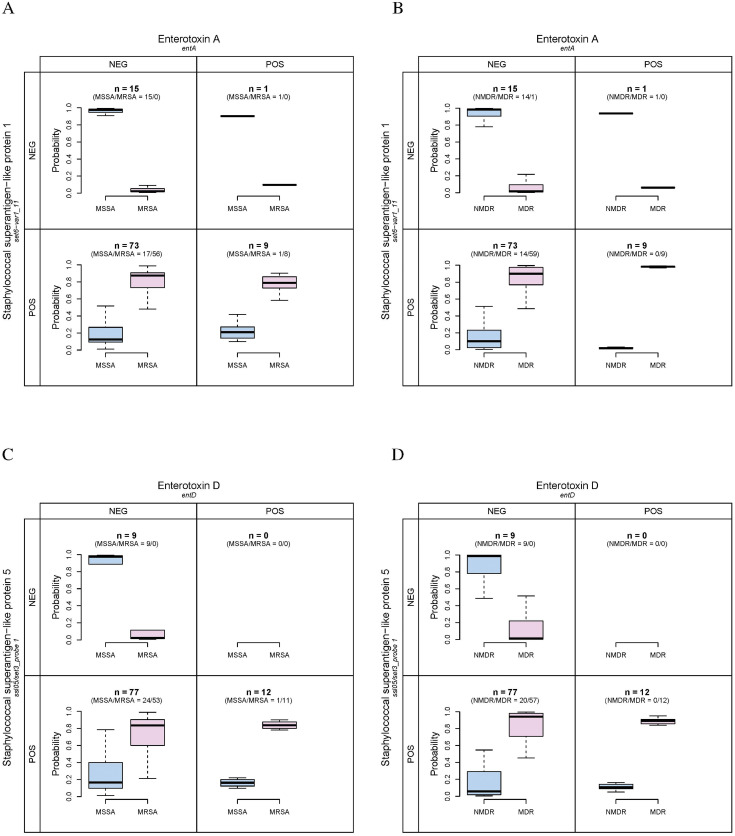
Interactions between staphylococcal enterotoxins A and D and superantigen-like proteins 1 and 5 for predicting the phenotypic expressions of MRSA and MDR *S. aureus*. The numbers of detected MSSA, MRSA, NMDR and MDR isolates are listed in the parentheses. A: The interaction between *entA* and *set6-var1_11* for MRSA. B: The interaction between *entA* and *set6-var1_11* for MDR *S. aureus*. C: The interaction between *entD* and *ssl05/set3_probe 1* for MRSA. D: The interaction between *entD* and *ssl05/set3_probe 1* for MDR *S. aureus*.

The interaction detection results for all three outcomes are very similar. Enterotoxins strongly interact with staphylococcal superantigen-like proteins. Enterotoxin A, B, and *egc* cluster interact with staphylococcal superantigen-like protein 1. As shown in Fig 1A for the genotypic expression of MRSA, when both *entA* and *set6-var1_11* are negative, the predicted probability of MRSA is much higher than MSSA (range 90.13%—99.06% for MSSA and 0.94%—9.87% for MRSA, n = 15). When both are positive, the predicted probability of MSSA is much lower than MRSA (range 5.69%—41.25% for MSSA and 58.75%—94.31% for MRSA, n = 9). For the phenotypic expressions of MRSA and MDR *S. aureus*, these results are similar. As shown in Fig 2A, when both *entA* and *set6-var1_11* are negative, the predicted probability of MSSA is much higher than MRSA (range 86.06%—99.33% for MSSA and 0.67%—13.94% for MRSA, n = 15). When both are positive, the predicted probability of MSSA is much lower than MRSA (range 9.86%—41.63% for MSSA and 58.37%—90.14% for MRSA, n = 9). The result for MDR *S. aureus* is shown in Fig 2B: when both *entA* and *set6-var1_11* are negative, the predicted probability of NMDR is much higher than MDR (range 45.92%—99.73% for NMDR and 0.27%—54.08% for MDR, n = 15). When both are positive, the predicted probability of NMDR is much lower than MDR (range 0.98%—29.14% for NMDR and 70.86%—99.02% for MDR, n = 9).

As shown in Table 1, the percentage of enterotoxin B positive is similar for different MRSA and MDR statuses. Enterotoxin B possibly interacts with staphylococcal superantigen-like protein 1. The presence of enterotoxin B predicts higher probability of MDR or MRSA only when *ssl01/set6 (COL)* is positive. If *ssl01/set6 (COL)* is negative, *entB* positive is associated with higher probability of NMDR or MSSA. The result for the genotypic expression of MRSA is shown in Fig 1B. A similar interaction exists for *egc (total)* and *ssl01/set6 (Mu50+N315)*. As shown in Fig 1C, when both of them are negative, the predicted probability of MSSA is much higher than MRSA (range 86.26%—98.98% for MSSA and 1.02%—13.74% for MRSA, n = 8). When both are positive, the predicted probability of MSSA is much lower than MRSA (range 1.98%—76.21% for MSSA and 23.79%—98.02% for MRSA, n = 48). When the two genes are both positive or negative for these two interactions, the range for the probability of MDR *S. aureus* or the genotypic expression of MRSA is much overlap with its non-resistant counterpart, indicating that the interaction effects are weaker than the pair of *entA* and *set6-var1_11*. However, for the phenotypic expression of MRSA, the interaction between *egc (total)* and *ssl01/set6 (Mu50+N315)* is strong: when both of them are negative, the predicted probability of MSSA is much higher than MRSA (range 86.06%—98.65% for MSSA and 1.35%—13.94% for MRSA, n = 8). When both are positive, the predicted probability of MSSA is much lower than MRSA (range 1.19%—55.54% for MSSA and 44.46%—98.81% for MRSA, n = 48).

An interaction between enterotoxin D and staphylococcal superantigen-like protein 5 exists. For the prediction of the genotypic expression of MRSA, the result is shown in Fig 1D: when both *entD* and *ssl05/set3_probe 1* are negative, the predicted probability of MSSA is much higher than MRSA (range 65.98%—99.06% for MSSA and 0.94%—34.02% for MRSA, n = 9). When both are positive, the predicted probability of MSSA is much lower than MRSA (range 4.85%—46.4% for MSSA and 53.6%—95.15% for MRSA, n = 12). For the phenotypic expressions of MDR and MRSA, these results are similar. As shown in Fig 2C, when both *entD* and *ssl05/set3_probe 1* are negative, the predicted probability of MSSA is much higher than MRSA (range 51.85%—99.33% for MSSA and 0.67%—48.15% for MRSA, n = 9). When both are positive, the predicted probability of MSSA is much lower than MRSA (range 9.88%—47.17% for MSSA and 52.83%—90.12% for MRSA, n = 12). The result for MDR *S. aureus* is shown in Fig 2D: when both *entD* and *ssl05/set3_probe 1* are negative, the predicted probability of NMDR is much higher than MDR (range 48.54%—99.73% for NMDR and 0.27%—51.46% for MDR, n = 9). When both are positive, the predicted probability of NMDR is much lower than MDR (range 4.97%—30.95% for NMDR and 69.05%—95.03% for MDR, n = 12).

The interactions between enterotoxins are weaker. We found two interactions: enterotoxins A and Q, and enterotoxins D and R. When these genes present in pairs, the probability of MDR and MRSA is high. Take the genotypic expression of MRSA for example. As shown in S2 Fig, the predicted probability of MRSA is quite high when both enterotoxins A and Q are positive (range 58.75%—94.31%, n = 7). Similarly, when enterotoxins D and R are positive, the predicted probability of MRSA is high, ranging from 53.6%—95.15% in 11 isolates. However, the absence of enterotoxins A and Q or enterotoxins D and R in pairs does not indicate that the probability of MDR or MRSA is lower than its non-resistant counterpart, which means that these enterotoxins alone could not distinguish any outcome deterministically.

## Discussion

The recovery of MDR strains of *S. aureus* gave a warning to the usage of antibiotics. Methicillin and multidrug resistant *S. aureus* has high clinical significance and poses a potential public health hazard. We analyzed staphylococcal superantigen and enterotoxin genes to examine their potential for bacterial pathogenicity and probe their potential mechanisms of resistance to antibiotics. We found that enterotoxin D and staphylococcal superantigen-like proteins 5 are the most predominant virulence factors associated with MRSA, while enterotoxin D and staphylococcal superantigen-like proteins 1 are the most predominant virulence factors associated with MDR *S. aureus*. The presence of *entA* and *set6-var1_11*, as well as *entD* and *ssl05/set3_probe 1* in pairs, predicts MDR and MRSA. This, along with our findings of enterotoxins A, J, R and staphylococcal superantigen-like proteins 1, 3, 7, and 10 could evoke new research about their roles in the initiation and pathogenesis of the disease. Although staphylococcal enterotoxins were found to be highly correlated with MRSA, we found that they are more predictive for MDR status in ocular isolates. Since the patterns of staphylococcal enterotoxin genes are probably source-associated, identifying these genes is a potential method to trace the sources of infection in ocular infection. Clinically these findings are important because they allow us to determine the superantigen profile of persistent strains and may bring inspiration into the routine application of antimicrobial susceptibility testing to prevent the emergence of antibiotic-resistant strains of potential public health concern. Therefore, our results provide important antimicrobial resistance and hygienic information on the control and screening of *S. aureus* in ocular medicine.

The high accuracy of our machine learning Random Forest model indicates that there are significant differences in the spectrum of ocular pathology between different methicillin and multidrug resistant statuses. Since *S. aureus* isolates with the *mecA* gene are more likely to be MDR and difficult to treat, our model is valuable for facilitating optimal treatments of *S. aureus* ocular infections. Overall, staphylococcal superantigen-like proteins correlate with MRSA stronger than enterotoxins. This finding is similar to a previous study of livestock-associated MRSA isolates from farms and farmers with hospital-acquired MRSA [45]; the sources of our isolates are different, and we included more virulence factors to choose the most informative ones more selectively through the Random Forest model. We found that the information from staphylococcal superantigen-like proteins is redundant because 30 variables showed significant association with the genotypic expression of MRSA from 45 Pearson’s Chi-squared tests, but we only discovered 11 significant variables from our classification model. These 11 variables, including *set6-var1_11, set6-var2_11, ssl01/set6 (MW2+MSSA476), ssl03/set8_probe 1, ssl05/set3_probe 1, ssl05/set3 (RF122, probe-611), ssl05/set3_probe 2 (612), ssl05/set3 (MRSA252), ssl07/set1 (MRSA252)*, ssl07/set1 (AF188836), and *ssl10/set4*, cover staphylococcal superantigen-like proteins 1, 3, 5, 7, and 10, which indicates that staphylococcal superantigen-like proteins 2, 4, 6, 8, 9, 11 and staphylococcal exotoxin-like protein second locus could be highly correlated with these 11 virulence genes. Staphylococcal superantigen-like protein 6 seems not associated with MDR or MRSA.

Enterotoxin B was found to be more prevalent in MSSA than MRSA; whether such association is significant [23, 24] or not [25] is unclear. We found that this association was stronger when *ssl01/set6 (COL)* was negative. Our findings for enterotoxins A to D are similar to the results of isolates from urinary tract infections in Baba-Moussa et al. study [25] that enterotoxins A and D are more informative than enterotoxins B and C. After adjusting for other virulence factors, our model suggested that *entA (N315) / entP* positive indicated a higher probability of MRSA; the main effects of other enterotoxins were not as strong as *entD*. In other studies, enterotoxin genes demonstrated heterogeneous main effects [24, 46–48]. We believe that the complex interactions between enterotoxin genes and staphylococcal superantigen-like proteins contribute to these heterogeneous findings and potentially explain some temporal or geographic variation in the MDR and MRSA epidemic. Moreover, treatment with staphylococcal enterotoxin B has been used to suppress immune rejection during corneal transplantation in mice potentially due to its effects on T-cell depletion and acquiring donor-specific immunosuppression [49]. Our study inspires future research on staphylococcal enterotoxin B treatment for preventing resistance and maintaining the effectiveness of antibiotics.

Enterotoxin genes are more informative to predict MDR *S. aureus* compared with MRSA. The interactions between enterotoxin genes exist, but enterotoxins alone could not distinguish MRSA versus MSSA or NMDR versus MDR deterministically. For example, the predicted probability of MDR and MRSA is quite high when both enterotoxins A and Q or both enterotoxins D and R are positive. However, if these virulence factors were not produced, the probability of MDR or MRSA was not significantly lower. These results indicate an association between enterotoxins and *S. aureus* strains, regardless of the methicillin and multidrug resistance phenotype. However, when interacting with staphylococcal superantigen-like proteins, the effect of enterotoxins is deterministic. We found that when both *entA* and *set6-var1_11*, or both *egc (total)* and *ssl01/set6 (Mu50+N315)*, or both *entD* and *ssl05/set3_probe 1* are negative in pairs, the probability of MDR and MRSA is much lower than NMDR and MSSA, while the presence of these genes in pairs is associated MDR and MRSA. These genes may act in pairs or groups to exert profound toxic effects upon the immune system. Although it is unclear whether these combinations link to aggravation of the corneal damage or exacerbation of the ocular surface inflammatory response, these results provide valuable insight to effective treatments of *S. aureus* ocular infections and strict hygiene as well as preventative measures. Further research is needed to determine why the interaction between staphylococcal superantigens and enterotoxins is so strong.

We analyzed virulence factors in a more inclusive fashion to identify the complex interactions between virulence genes and find the ones that are the most informative to predict each outcome after adjusting for multiple predictors. It is possible that after adjustment for different virulence factors, the discoveries of informative virulence genes could be more consistent and reproducible. When the variables are multifactorial and interacted, a large sample size and flexible statistical assumption of the prediction model are often required. For this dataset, we tried classical logistic regression and lasso penalized logistic regression. None of the coefficients were significant from the classical logistic regression, and the lasso penalized logistic regression did not predict the outcome as accurately as the Random Forest model. Moreover, when the virulence genes are highly correlated and interacted, nonparametric variable important index [39, 50–52], instead of odds ratio or regression coefficient, may be more suitable for providing insights into ocular pathology. In looking back, we can now see that the success of the Random Forest model can be largely attributed to its ability to accommodate interactions. It delivered an ideal prediction accuracy and has a high potential for practical implementation. We demonstrated that combining the results of MDR and MRSA testing and staphylococcal enterotoxin and superantigen profiling is feasible in Random Forest models for the discrimination of the genetic diversity and drug resistance in *S. cereus*.

## Supporting information

S1 Table Variable importance results for predicting the genotypic expression of MRSA (full version of Table 2). (DOCX)

S2 Table Summary of duplicated results of S1 Table. (DOCX)

S3 Table Role of *mecA* genotype in the phenotypic expressions of methicillin and multidrug resistance. (DOCX)

S4 Table Informative virulence genes for predicting the phenotypic expression of MRSA. (DOCX)

S5 Table Informative virulence genes for predicting the phenotypic expression of MDR *S. aureus*. (DOCX)

S1 Fig Adjusted probability of the genotypic expression of MRSA from Random Forest prediction plotted against candidate virulence genes. A: Higher prevalence of enterotoxin P in MRSA isolates after adjusting for other virulence genes. In other words, *entA (N315) / entP* positive is associated with MRSA. B: After adjusting for other virulence genes, *ssl05/set3 (RF122, probe-611)* positive is associated with MRSA. C: After adjusting for other virulence genes, *ssl07/set1 (MRSA252)* negative is associated with MRSA.
(TIF)

S2 Fig Random Forest estimated probabilities of MSSA and MRSA in terms of genotypic expression plotted against selected interactions between enterotoxins. A: The interaction between *entQ* and *entA*. B: The interaction between *entR* and *entD*.
(TIF)
