# Supplementary material for: Interactions between staphylococcal enterotoxins A and D and superantigen-like proteins 1 and 5 for predicting methicillin and multidrug resistance profiles among Staphylococcus aureus ocular isolates
Source: PLoS One. 2021 Jul 28;16(7):e0254519. doi: 10.1371/journal.pone.0254519 (PMC8318242; doi:10.1371/journal.pone.0254519)
Supplement: S1 Table — (DOCX) [file pone.0254519.s001.docx]

**S1 Table. Variable importance results for predicting the genotypic expression of MRSA (full version of Table 2).**

|  |  | Number of | **Fisher’s exact test** | | **Random Forest** | | |
| --- | --- | --- | --- | --- | --- | --- | --- |
| Virulence factor | Virulence gene | NEG/POS  /AMB † | OR | 95% CI | Est | SE | Sig |
| Enterotoxin A | *entA* | 88/10 | 3.3 | 0.61-33.61 | 0.89 | 0.49 | * |
| Enterotoxin A, allele from strain 320E | *entA (320E)* | 98 |  |  |  |  |  |
| Enterotoxin A, allele from strain  N315 = enterotoxin P | *entA (N315) / entP* | 90/8 | 0.73 | 0.13-4.2 | 1.09 | 0.52 | * |
| Enterotoxin B | *entB* | 86/12 | 1.06 | 0.26-4.58 | -0.1 | 0.19 |  |
| Enterotoxin C | *entC* | 94/4 | 0.24 | 0-3.11 | -0.09 | 0.14 |  |
| Enterotoxin D | *entD* | 86/12 | 4.29 | 0.84-42.53 | 3.1 | 0.98 | *** |
| Enterotoxin E | *entE* | 98 |  |  |  |  |  |
| Enterotoxin G | *entG* | 40/58 | 1.37 | 0.56-3.36 | -0.04 | 0.16 |  |
| Enterotoxin H | *entH* | 96/2 | 0 | 0-3.97 | 0.02 | 0.02 |  |
| Enterotoxin I | *entI* | 40/58 | 1.37 | 0.56-3.36 | -0.04 | 0.16 |  |
| Enterotoxin J | *entJ* | 83/15 | 1.6 | 0.45-6.51 | 0.77 | 0.32 | ** |
| Enterotoxin K | *entK* | 76/22 | 2.38 | 0.78-8.26 | 0.17 | 0.67 |  |
| Enterotoxin L | *entL* | 94/4 | 0.24 | 0-3.11 | -0.09 | 0.14 |  |
| Enterotoxin M | *entM* | 40/58 | 1.37 | 0.56-3.36 | -0.04 | 0.16 |  |
| Enterotoxin N | *entN (cons)* | 40/58 | 1.37 | 0.56-3.36 | -0.04 | 0.16 |  |
|  | *entN (other than RF122)* | 40/58 | 1.37 | 0.56-3.36 | -0.04 | 0.16 |  |
| Enterotoxin O | *entO* | 40/58 | 1.37 | 0.56-3.36 | -0.04 | 0.16 |  |
| *egc* cluster | *egc (total)* | 40/58 | 1.37 | 0.56-3.36 | -0.04 | 0.16 |  |
| Enterotoxin Q | *entQ* | 76/22 | 2.38 | 0.78-8.26 | 0.17 | 0.67 |  |
| Enterotoxin R | *entR* | 84/14 | 2.05 | 0.54-9.68 | 1.78 | 0.63 | ** |
| Enterotoxin U and/or Y | *entU* | 40/58 | 1.37 | 0.56-3.36 | -0.04 | 0.16 |  |
| Staphylococcal superantigen-like protein 1 | *set6-var1_11* | 16/82 | Inf | 7.39-Inf | 1.8 | 0.41 | *** |
|  | *set6-var2_11* | 81/14/3 | 0 | 0-0.17 | 1.07 | 0.32 | *** |
|  | *set6-var1_12* | 15/72/11 | 7.8 | 1.87-47.1 | 1.02 | 0.63 |  |
|  | *set6-var2_12* | 92/4/2 | 0 | 0-1.09 | -0.04 | 0.19 |  |
|  | *set6-var4_11* | 7/91 | 8.98 | 1.02-428.15 | 0.08 | 0.09 |  |
|  | *ssl01-RF122* | 95/3 | 0 | 0-1.78 | 0.04 | 0.03 |  |
|  | *ssl01/set6 (COL)* | 67/31 | 1.91 | 0.73-5.27 | 0.19 | 0.19 |  |
|  | *ssl01/set6 (Mu50+N315)* | 18/49/31 | 36.47 | 4.89-1640.53 | 0.94 | 0.63 |  |
|  | *ssl01/set6 (MW2+MSSA476)* | 94/4 | 0 | 0-1.1 | 0.14 | 0.06 | ** |
|  | *ssl01/set6 (MRSA252)* | 94/4 | 0 | 0-1.1 | 0 | 0.01 |  |
|  | *ssl01/set6 (RF122)* | 97/1 | 0 | 0-29.25 | 0 | 0 |  |
|  | *ssl01/set6 (other alleles)* | 89/9 | 0.08 | 0-0.63 | 0.03 | 0.09 |  |
| Staphylococcal superantigen-like protein 2 | *ssl02/set7* | 5/90/3 | Inf | 1.4-Inf | 0.09 | 0.08 |  |
|  | *ssl02/set7 (MRSA252)* | 26/7/65 | 0 | 0-0.66 | 0.35 | 0.45 |  |
| Staphylococcal superantigen-like protein 3 | *ssl03/set8_probe 1* | 7/91 | Inf | 2.12-Inf | 0.07 | 0.04 | * |
|  | *ssl03/set8_probe 2* | 7/91 | Inf | 2.12-Inf | 0.07 | 0.04 | * |
|  | *ssl03/set8 (MRSA252, SAR0424)* | 96/2 | 0 | 0-3.97 | 0 | 0 |  |
| Staphylococcal superantigen-like protein 4 | *ssl04/set9* | 8/90 | Inf | 2.58-Inf | 0.05 | 0.09 |  |
|  | *ssl04/set9 (MRSA252, SAR0425)* | 87/7/4 | 0 | 0-0.48 | 0.15 | 0.17 |  |
| Staphylococcal superantigen-like protein 5 | *ssl05/set3_probe 1* | 9/89 | Inf | 3.06-Inf | 0.35 | 0.16 | * |
|  | *ssl05/set3 (RF122, probe-611)* | 12/8/78 | 0 | 0-3.54 | 1.71 | 0.46 | *** |
|  | *ssl05/set3_probe 2 (612)* | 7/83/8 | Inf | 2.71-Inf | 2.64 | 0.55 | *** |
|  | *ssl05/set3 (MRSA252)* | 91/7 | 0 | 0-0.47 | 0.08 | 0.05 | * |
| Staphylococcal superantigen-like protein 6 | *ssl06/set21* | 60/38 | 0.74 | 0.3-1.83 | -0.3 | 0.24 |  |
|  | *ssl06 (NCTC8325+MW2)* | 47/41/10 | 0.6 | 0.23-1.52 | 0.06 | 0.34 |  |
| Staphylococcal superantigen-like protein 7 | *ssl07/set1* | 6/91/1 | Inf | 1.74-Inf | 0.02 | 0.05 |  |
|  | *ssl07/set1 (MRSA252)* | 5/2/91 | 0 | 0-Inf | 0.18 | 0.1 | * |
|  | *ssl07/set1 (AF188836)* | 65/5/28 | 0 | 0-0.71 | 2.13 | 0.84 | ** |
| Staphylococcal superantigen-like protein 8 | *ssl08/set12_probe 1* | 7/91 | Inf | 2.12-Inf | 0.07 | 0.04 | * |
|  | *ssl08/set12_probe 2* | 7/91 | Inf | 2.12-Inf | 0.07 | 0.04 | * |
| Staphylococcal superantigen-like protein 9 | *ssl09/set5_probe 1* | 10/88 | 3.49 | 0.74-22.3 | -0.2 | 0.11 |  |
|  | *ssl09/set5_probe 2* | 10/88 | 3.49 | 0.74-22.3 | -0.2 | 0.11 |  |
|  | *ssl09/set5 (MRSA252)* | 91/7 | 0 | 0-0.47 | 0.08 | 0.05 | * |
| Staphylococcal superantigen-like protein 10 | *ssl10/set4* | 11/87 | Inf | 4.11-Inf | 0.41 | 0.15 | ** |
|  | *ssl10 (RF122)* | 82/4/12 | 0 | 0-1.1 | -0.05 | 0.34 |  |
|  | *ssl10/set4 (MRSA252)* | 4/6/88 | 0 | 0-26 | 0.4 | 0.46 |  |
| Staphylococcal superantigen-like protein 11 | *ssl11/set2 (COL)* | 67/31 | 1.91 | 0.73-5.27 | 0.19 | 0.19 |  |
|  | *ssl11+set2(Mu50+N315)* | 50/48 | 3.05 | 1.24-7.8 | 0.12 | 0.2 |  |
|  | *ssl11+set2(MW2+MSSA476)* | 95/3 | 0 | 0-1.78 | 0.05 | 0.04 |  |
|  | *ssl11/set2 (MRSA252)* | 92/6 | 0 | 0-0.59 | 0.03 | 0.04 |  |
| Staphylococcal exotoxin-like protein, | *setB3* | 7/91 | Inf | 2.12-Inf | 0.07 | 0.04 | * |
| second locus | *setB3 (MRSA252)* | 91/7 | 0 | 0-0.47 | 0.08 | 0.05 | * |
|  | *setB2* | 7/91 | Inf | 2.12-Inf | 0.07 | 0.04 | * |
|  | *setB2 (MRSA252)* | 91/7 | 0 | 0-0.47 | 0.08 | 0.05 | * |
|  | *setB1* | 2/94/2 | Inf | 0.26-Inf | 0 | 0.01 |  |
| Sources | *sources* | 17/1/76/  1/1/2 | 0.13 | 0.01-0.61 | -0.36 | 0.45 |  |

† Numbers of different levels are listed in the order of positive (POS), negative (NEG), and ambiguous (AMB); the number of AMB is omitted if there are no AMB isolates. For isolate sources listed in the bottom, the counts of levels are listed in the order of conjunctiva, conjunctiva/lids, cornea, lids, socket, and suture; its odds ratio compares cornea (as POS) versus conjunctiva (as NEG) valued smaller than one, indicating that fewer MRSA isolates are collected from cornea than conjunctiva.

OR indicates odds ratio, which is used to indicate the direction of the correlation: if the value is larger than one, the virulence gene is more likely to be detected (as more POS isolates) in MRSA isolates. Inf indicates infinity value since some entry of the contingency table is zero.

Est indicates estimated VIMP from random forest model in terms of contribution to classification accuracy in percentage; SE indicates standard error; Sig indicates significant level according to *P* values: * for *p* $\leq$ 0.05; ** for *p* $\leq$ 0.01; *** for *p* $\leq$ 0.001.
