# Supplementary material for: Interactions between staphylococcal enterotoxins A and D and superantigen-like proteins 1 and 5 for predicting methicillin and multidrug resistance profiles among Staphylococcus aureus ocular isolates
Source: PLoS One. 2021 Jul 28;16(7):e0254519. doi: 10.1371/journal.pone.0254519 (PMC8318242; doi:10.1371/journal.pone.0254519)
Supplement: S2 Table — (DOCX) [file pone.0254519.s002.docx]

**S2 Table. Summary of duplicated results of S1 Table.**

| Virulence gene | Virulence gene(s) with the same detection result | | | | | | |
| --- | --- | --- | --- | --- | --- | --- | --- |
| *egc (total)* | *entI* | *entM* | *entN (cons)* | *entN*  *(other than RF122)* | *entO* | *entG* | *entU* |
| *entC* | *entL* |  |  |  |  |  |  |
| *entK* | *entQ* |  |  |  |  |  |  |
| *ssl03/set8_probe 1* | *ssl03/set8_probe 2* | *ssl08/set12_probe 1* | *ssl08/set12_probe 2* | *setB3* | *setB2* |  |  |
| *ssl09/set5_probe 1* | *ssl09/set5_probe 2* |  |  |  |  |  |  |
| *ssl05/set3 (MRSA252)* | *ssl09/set5 (MRSA252)* | *setB3 (MRSA252)* | *setB2 (MRSA252)* | |  |  |  |
| *ssl01/set6 (COL)* | *ssl11/set2 (COL)* |  |  |  |  |  |  |
