# Supplementary material for: Interactions between staphylococcal enterotoxins A and D and superantigen-like proteins 1 and 5 for predicting methicillin and multidrug resistance profiles among Staphylococcus aureus ocular isolates
Source: PLoS One. 2021 Jul 28;16(7):e0254519. doi: 10.1371/journal.pone.0254519 (PMC8318242; doi:10.1371/journal.pone.0254519)
Supplement: S3 Table — (DOCX) [file pone.0254519.s003.docx]

**S3 Table. Role of *mecA* genotype in the phenotypic expressions of methicillin and multidrug resistance.**

| Phenotype | | *mecA* genotype | |
| --- | --- | --- | --- |
| MDR | MRSA | Negative | Positive |
| No | No | 25 | 0 |
|  | Yes | 0 | 4 |
| Yes | No | 9 | 0 |
|  | Yes | 8† | 52  † The *mecA* gene was not detected because the test methods were varied, and microarray was done after the phenotypic assays --- hence freezing may impact the stability of *mecA*. |
