# Supplementary material for: Interactions between staphylococcal enterotoxins A and D and superantigen-like proteins 1 and 5 for predicting methicillin and multidrug resistance profiles among Staphylococcus aureus ocular isolates
Source: PLoS One. 2021 Jul 28;16(7):e0254519. doi: 10.1371/journal.pone.0254519 (PMC8318242; doi:10.1371/journal.pone.0254519)
Supplement: S5 Table — (DOCX) [file pone.0254519.s005.docx]

|  |  | **Fisher’s exact test** | | **Random Forest** | | |
| --- | --- | --- | --- | --- | --- | --- |
| Virulence factor | Virulence gene | OR | 95% CI | Est | SE | Sig |
| enterotoxin C | *entC* | 0 | 0-0.6 | 0.54 | 0.23 | ** |
| enterotoxin D | *entD* | Inf | 1.29-Inf | 3.63 | 1.01 | *** |
| enterotoxin J | *entJ* | 1.81 | 0.44-10.86 | 0.39 | 0.18 | * |
| enterotoxin R | *entR* | 1.64 | 0.39-9.89 | 0.49 | 0.22 | * |
| staphylococcal superantigen-like protein 1 | *set6-var1_11* | 68.64 | 9.24-3050.07 | 0.78 | 0.27 | ** |
|  | *set6-var4_11* | 17.16 | 1.93-823.59 | 0.37 | 0.16 | * |
|  | *ssl01/set6 (Mu50+N315)* | 78.94 | 9.96-3632.26 | 2.27 | 0.48 | *** |
|  | *ssl01/set6 (other alleles)* | 0 | 0-0.17 | 0.44 | 0.15 | ** |
| staphylococcal superantigen-like protein 4 | *ssl04/set9* | Inf | 4.98-Inf | 0.14 | 0.06 | ** |
| staphylococcal superantigen-like protein 5 | *ssl05/set3_probe 1* | Inf | 6-Inf | 0.55 | 0.17 | *** |
|  | *ssl05/set3 (RF122, probe-611)* | 0 | 0-1.39 | 0.63 | 0.22 | ** |
|  | *ssl05/set3_probe 2 (612)* | Inf | 6.05-Inf | 1.81 | 0.49 | *** |
| staphylococcal superantigen-like protein 10 | *ssl10/set4* | Inf | 8.35-Inf | 0.5 | 0.17 | ** |
|  | *ssl10 (RF122)* | 0 | 0-0.51 | 0.58 | 0.26 | * |

**S5 Table. Informative virulence genes for predicting the phenotypic expression of MDR *S. aureus*.**

OR indicates odds ratio, which is used to indicate the direction of the correlation: if the value is larger than one, the virulence gene is more likely to be detected (as more positive isolates) in MDR isolates; if the value is smaller than one, the virulence gene is less likely to be detected (as more negative isolates) in MDR isolates. Inf indicates infinity value since some entry of the contingency table is zero.

Est indicates estimated VIMP from Random Forest model in terms of contribution to classification accuracy in percentage; SE indicates standard error; Sig indicates significant level according to *P* values: * for *p* $\leq$ 0.05; ** for *p* $\leq$ 0.01; *** for *p* $\leq$ 0.001.
