# Supplementary figures and images for: Interactions between staphylococcal enterotoxins A and D and superantigen-like proteins 1 and 5 for predicting methicillin and multidrug resistance profiles among Staphylococcus aureus ocular isolates
Source: PLoS One. 2021 Jul 28;16(7):e0254519. doi: 10.1371/journal.pone.0254519 (PMC8318242; doi:10.1371/journal.pone.0254519)

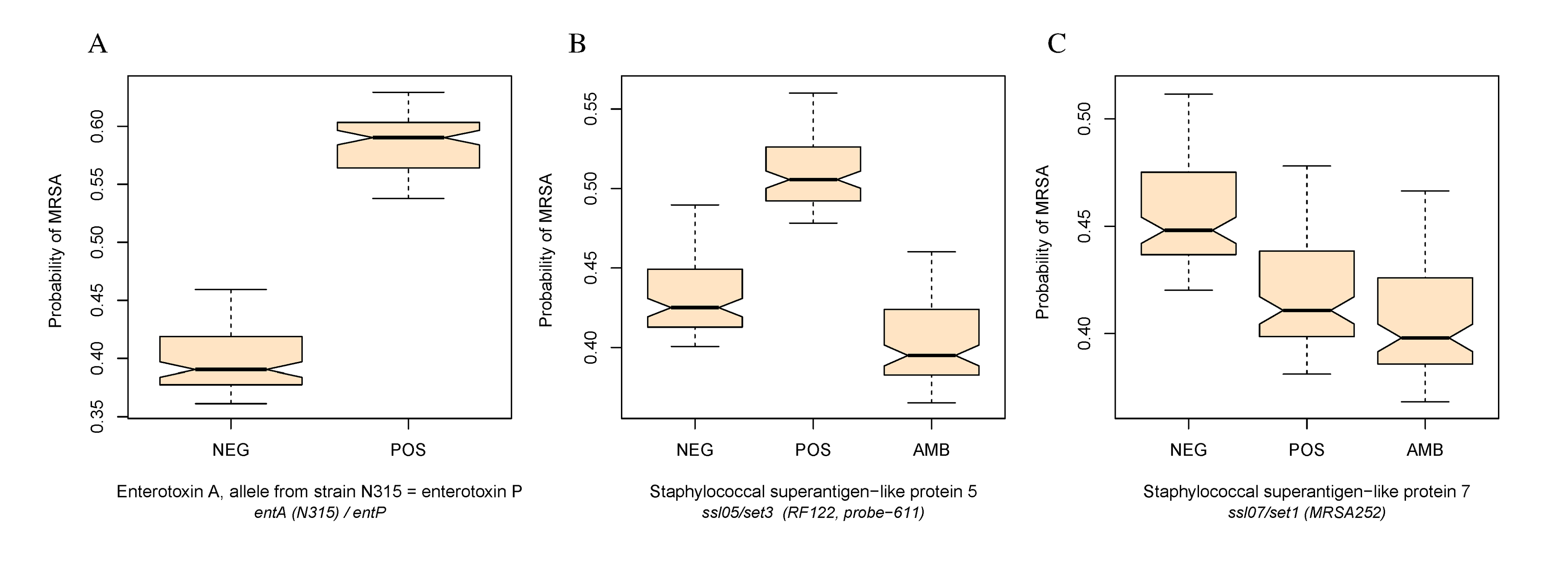

Supplement: S1 Fig — A: Higher prevalence of enterotoxin P in MRSA isolates after adjusting for other virulence genes. In other words, entA (N315) / entP positive is associated with MRSA. B: After adjusting for other virulence genes, ssl05/set3 (RF122, probe-611) positive is associated with MRSA. C: After adjusting for other virulence genes, ssl07/set1 (MRSA252) negative is associated with MRSA. (TIF) [file pone.0254519.s006.tif]

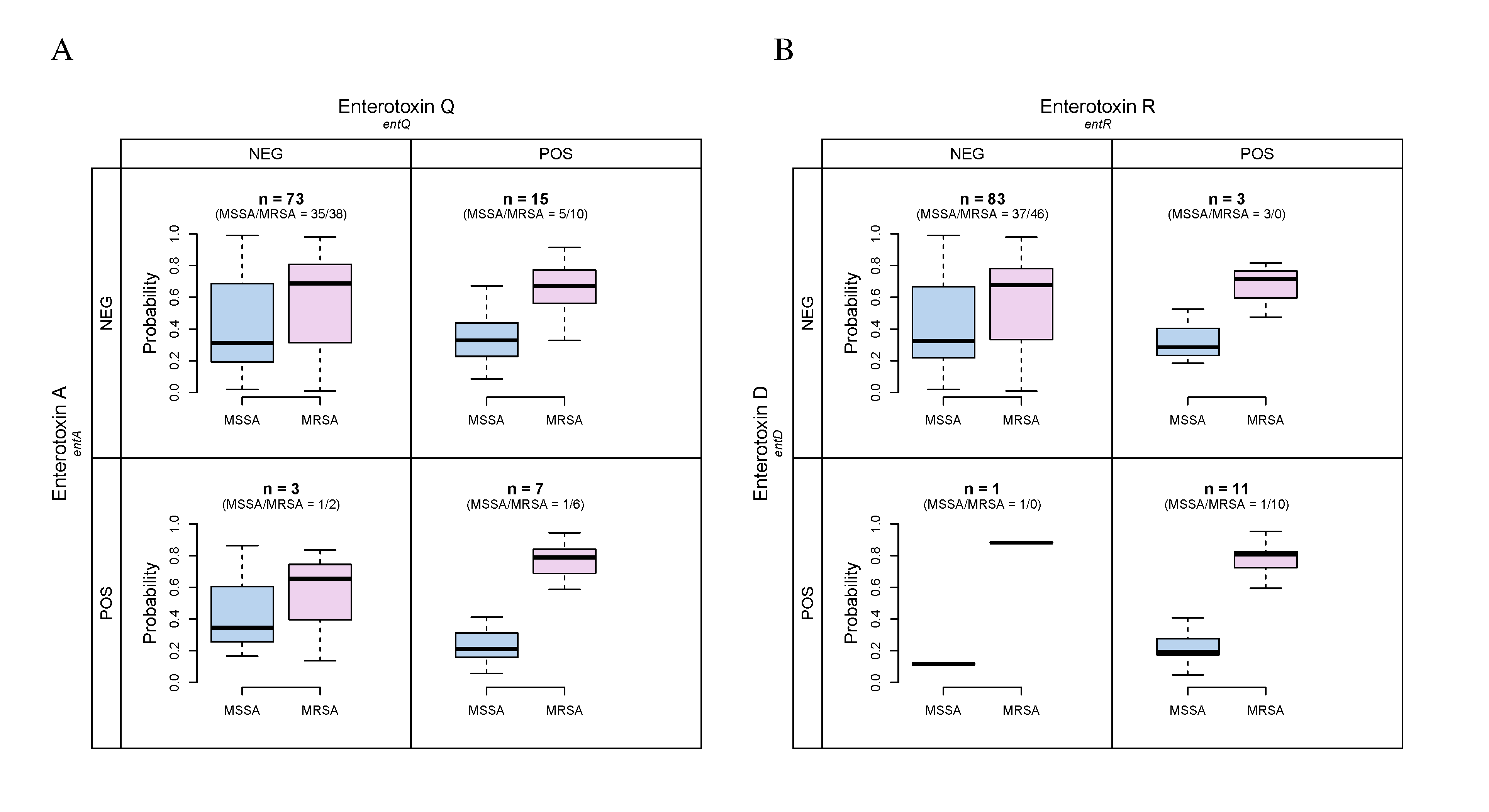

Supplement: S2 Fig — A: The interaction between entQ and entA. B: The interaction between entR and entD. (TIF) [file pone.0254519.s007.tif]
